# Incidence of HER2-expressing brain metastases in patients with HER2-null breast cancer: a matched case analysis

**DOI:** 10.1038/s41523-023-00592-5

**Published:** 2023-10-23

**Authors:** Nelson S. Moss, Jolene M. Singh, Anne S. Reiner, Joshua Z. Drago, Shanu Modi, Andrew D. Seidman, Sarat Chandarlapaty, Dara S. Ross

**Affiliations:** 1 Memorial Sloan Kettering Cancer Center, Department of Neurosurgery and Brain Metastasis Center, New York, NY USA; 2 Memorial Sloan Kettering Cancer Center, Department of Epidemiology and Biostatistics, New York, NY USA; 3 Memorial Sloan Kettering Cancer Center, Breast Medicine Service, Department of Medicine, New York, NY USA; 4 Memorial Sloan Kettering Cancer Center, Department of Pathology, New York, NY USA

**Keywords:** Breast cancer, Diagnostic markers

## Abstract

The HER2-directed antibody-drug conjugate trastuzumab deruxtecan is active against lower levels of HER2 expression than prior-generation therapies. The rate of HER2 expression in brain metastases among patients with initially HER2-null breast cancer is undefined, and receptor discordance in advanced breast cancer with brain metastases may underestimate CNS response potential in the absence of brain metastasis sampling. In this cohort study including 136 patients with 401 samples scored according to ASCO/CAP guidelines, 15/28 patients (54%) with HER2-null primary breast cancer have detectable HER2 expression in subsequently resected brain metastases, a significant discordant population.

Brain metastases are a significant cause of neurologic morbidity and mortality in patients with human epidermal growth factor receptor 2 (HER2)-expressing breast cancer. HER2-directed antibody, antibody-drug conjugate and small molecule tyrosine kinase inhibitor-based treatment regimens are effective for patients with HER2+ (over-expressing) cancers, defined as a score of 3+ by immunohistochemistry (IHC; scale of 0–3 + ) and/or *ERBB2* copy number (CN) elevation by in situ hybridization (ISH; HER2/CEP17 ratio ≥2.0 or CN ≥ 6.0 signals/cell), and in some cases less effective for brain metastases owing to the blood-brain tumor barrier^1–3^. Recently, the antibody-drug conjugate trastuzumab deruxtecan (T-DXd) has shown efficacy in patients with lower HER2 expression levels (HER2-low; IHC1+ or IHC2 + /ISH-negative) in the DESTINY-Breast04 study^4^. While previous studies have shown discordance in HER2 expression levels along the HER2 + /HER2- spectrum between primary breast cancer specimens and matched brain metastases from the same patients, HER2-low activity and data supporting T-DXd efficacy in the CNS (for example in the TUXEDO-1 study demonstrating 73% best overall intracranial response rate)^5–8^ warrant investigation of the rate of HER2 discordance in patients with brain metastases along the full range of IHC detection.

Of 245 patients who underwent breast cancer brain metastasis resection from 2006-2022, 136 had ≥1 matched sample available; 401 total evaluable samples were included in the analysis, all of which were centrally reviewed at the same institution (365 [91%] initially were resected internally and 35 [9%] sampled externally with unstained slides subsequently processed and reviewed internally, except 1 case [<1%] for which external stained slides were reviewed internally). Of 133 samples (from 89 patients) with targeted sequencing, *ERBB2* mutations were identified in 6 samples (4.5%) from 4 patients; of these 3 were HER2-positive, 2 HER2-low (IHC 2 + /ISH-) and 1 HER2-null. Of 12 cases where targeted sequencing was available for ≥2 matched specimens, all had ≥1 shared mutation suggesting shared clonal origin of the metastasis. HER2 agreement (HER2-null versus HER2-expressing) was moderate (Kappa = 0.43; 95%CI = 0.23–0.64) for primary breast cancer versus brain metastasis (Fig. 1). Among patients with initially HER2-null disease, 15/28 (54%) had IHC scores of ≥ 1+ for their first brain metastasis, of which 2 subsequently were treated with (and 1 partially responded to) T-DXd. The brain metastases of 5/66 (7.6%) patients with HER2-expressing primary breast tumors were HER2-null. Among patients who had a change, gained expression was more common than lost expression (*p* = 0.03). Of note, approximately a third of primary brain metastases were HER2-low. For patients with multiple sampled brain metastases (*n* = 10), all had HER2-expressing primary brain metastasis and HER2-expressing subsequent brain metastases except for 1 patient (10%). HER2 agreement (HER2-null versus HER2-detectable) was moderate (Kappa = 0.50; 95%CI = 0.02–0.98) for primary breast cancer-lymph node metastasis pairs and fair for primary breast cancer-extracranial metastasis pairs (Kappa = 0.40; 95% CI = 0.0–0.73). For the 12 patients with 2+ extracranial metastases, and the 23 patients with 2+ profiled extracranial sites (including both extracranial metastases and lymph nodes), concordance for the first 2 sites were fair agreement only (kappa = 0.33, 95%CI: 0.0–0.84; and kappa = 0.38, 95%CI: 0.04–0.72), respectively. Time between primary and metastatic samples from all locations was not correlated with gain/loss of HER2 expression (Table 1, Fig. 2; OR = 1.002; 95%CI = 0.998–1.006; *p* = 0.37). The post hoc subset analyses of 10 random HER2 discordant and 10 random HER2 concordant breast-brain metastasis pairs demonstrated equivalent distributions of prior and intercurrent chemotherapy and radiation treatments. Finally, sensitivity analyses excluding patients with multiple and HER2 discordant primaries did not appreciably alter any results.

**Fig. 1:**
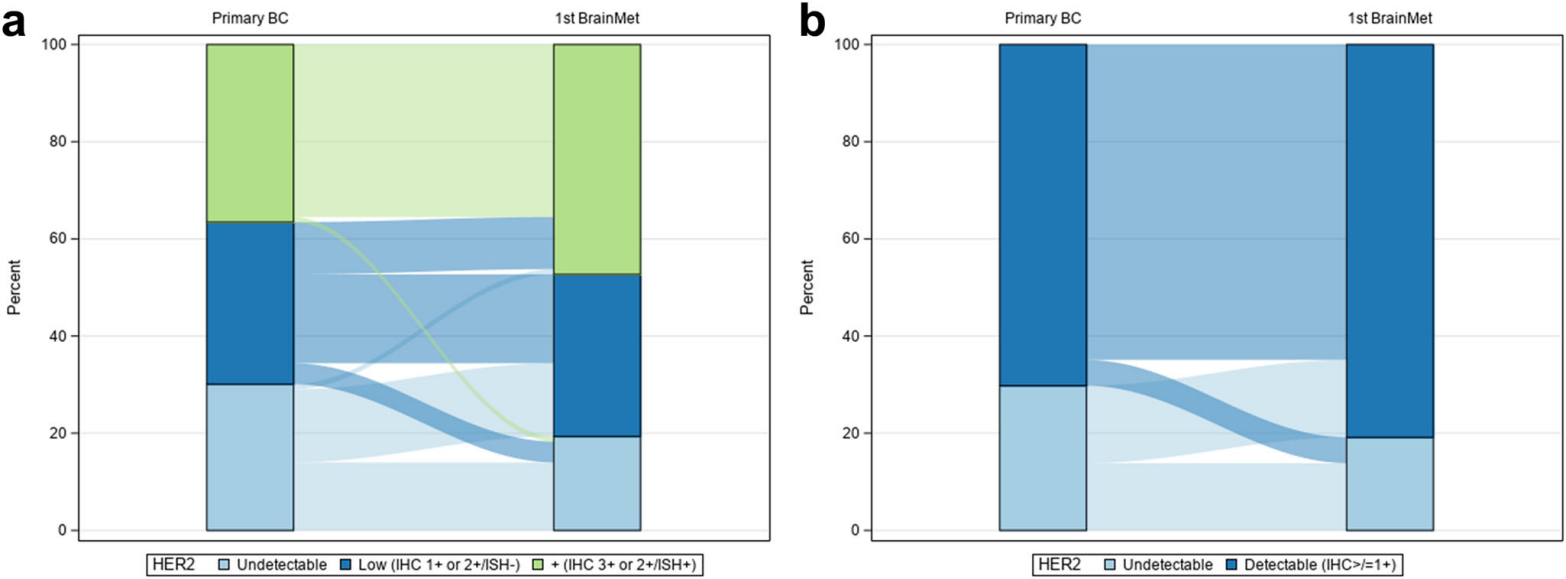
HER2 expression discordance in primary breast cancers and matched brain metastases. Analyses are stratified by HER2-null/-low/-positive (IHC0; 1+ or 2 + /ISH-; 2 + /ISH+ or 3 + , respectively; panel **a**), or HER2-undetectable/-detectable (IHC0 versus IHC > 0). Figure 1a comprises 93 patients and Fig. 1b comprises 94 patients. One case excluded from the former analysis was included in the latter as it was not classifiable to HER2-low or -positive. BC breast cancer.

**Table 1:** Primary breast cancer versus metastatic HER2 detectability.

Primary BC HER2 status	First brain metastasis status	N	%	Kappa (95% CI)
Null	Null	13	14	0.43 (0.23–0.64)
Null	Detectable	15	16	
Detectable	Null	5	5	
Detectable	Detectable	61	65	

Primary BC HER2 status	First LN status	N	%	
Null	Null	4	33	0.50 (0.02–0.98)
Null	Detectable	2	17	
Detectable	Null	1	8	
Detectable	Detectable	5	42	

Primary BC HER2 status	First extracranial metastasis status	N		
Null	Null	7	26	0.40 (0.07–0.73)
Null	Detectable	2	7	
Detectable	Null	6	22	
Detectable	Detectable	12	44	

*BC* breast cancer, *LN* lymph node. Null: IHC 0; Detectable: IHC ≥ 1 + .

**Fig. 2:**
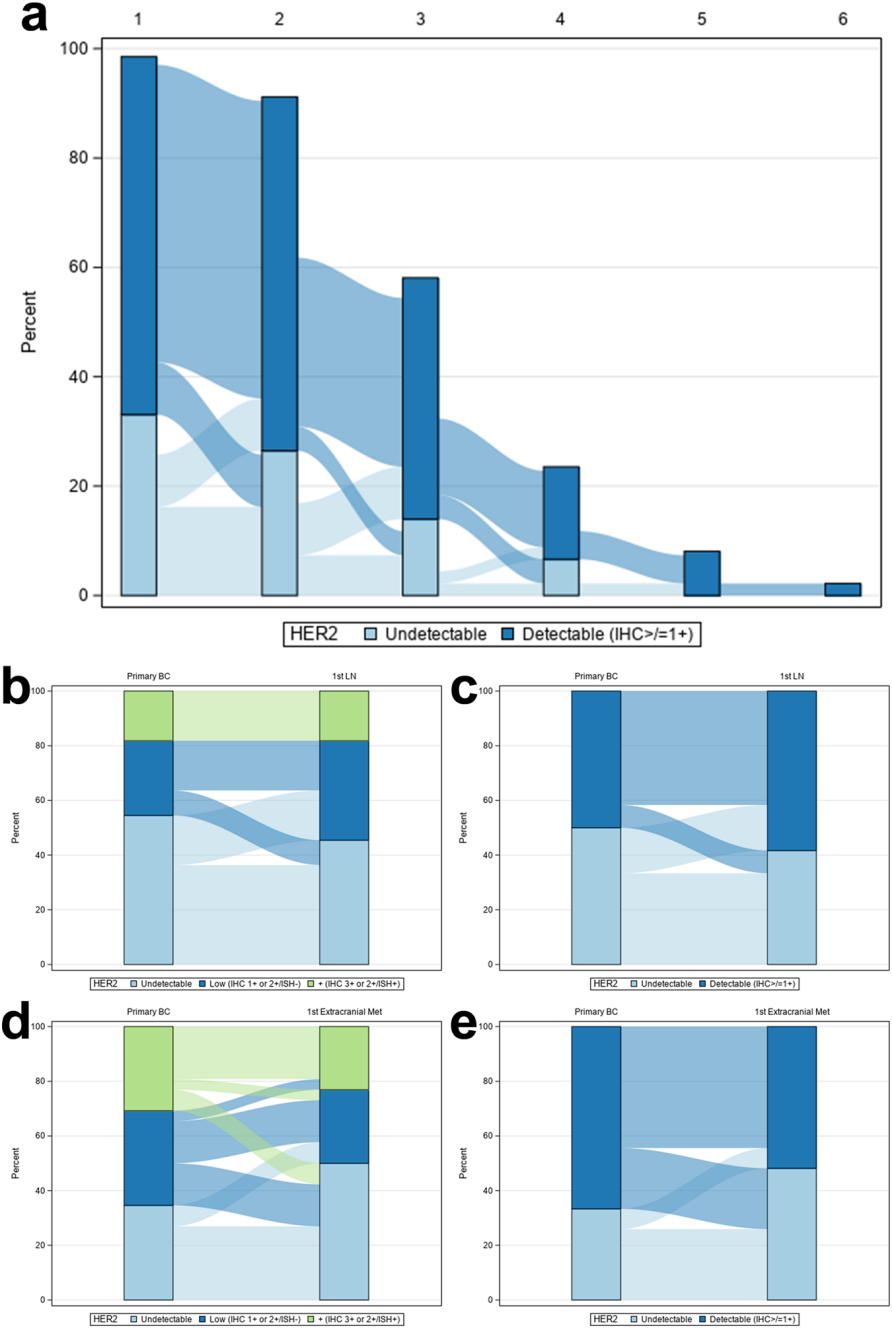
HER2 status by chronology and metastatic site. For chronologic analysis, the HER2 expression of all samples from patients with ≥1 brain metastasis and >1 extracranial sample were plotted longitudinally (**a**). Each bar is labeled 1 through 6 for the temporal sample number. There was no association between time between primary and metastatic samplings and gain/loss of HER2 expression (OR = 1.002; 95%CI = 0.998–1.006; *p* = 0.37). For patients with matched primary breast cancer and malignant lymph nodes (panels **b**, **c**) or extracranial metastases (panels **d**, **e**), HER2 expression concordance was plotted across the HER2-null/-low/-positive and -null/-detectable spectra as per Fig. 1. BC breast cancer, LN lymph node.

HER2 expression is heterogeneous and dynamic in breast and other cancers^9,10^. Brain metastases, which are both particularly refractory to systemic anticancer therapies, and a manifestation of advanced disease, are an urgent unmet need especially in HER2+ breast cancer given heavy dependence on antibody-based therapies which may have more limited CNS penetration than other anticancer drug classes. Until recently, HER2 + /- stratification as defined by ASCO/CAP guidelines was the only relevant clinical distinction given its correlation with response to legacy agents. Multiple studies have shown that a given patient’s brain metastases have an approximately 12% discordance with their primary tumor when stratifying HER2 in this binary, positive versus negative fashion, most commonly with increased expression in brain metastases^11,12^.

Our finding that when stratifying by HER2-null (IHC0) versus low or greater (IHC ≥ 1 + ), which is the key stratification for T-DXd, one in five patients have discordant HER2 expression in their first brain metastasis compared to their primary breast cancer. This may be due to a high degree of evolutionary selective pressure on these metastases particularly for patients with advanced disease.

Forthcoming data are expected to yield insight into T-DXd’s CNS-specific and extracranial efficacy in HER2-low and even HER2-null settings. Early data from the DAISY II study demonstrating T-DXd activity in 31% of patients with HER2-null advanced breast cancer raises the question of whether this agent acts altogether independently of HER2 binding, or whether more sensitive tissue measures of HER2 would further stratify response^13^. Furthermore, it is unclear whether T-DXd activity is dependent on HER2 expression to the same degree intracranially as extracranially. Such data will be needed to inform the utility of additional sampling, particularly in cases of CNS-only progression in T-DXd-naïve patients with otherwise HER2-undetectable disease, as compared to the known risks of CNS biopsy. Noninvasive biomarkers (e.g. molecular imaging and liquid biopsy) may also help fill this gap.

This study has several limitations. Firstly, HER2 expression can be heterogeneous across given tumors, introducing some risk of sampling error; however, the results reported were all from full-slide readouts and performed according to standardized guidelines. While HER2 IHC interrater concordance (≥70% agreement) in the 0-1+ range has been reported to be 75% using a standardized tissue microarray^14^, nearly all cases in this study were initially evaluated by a core group of subspecialized breast pathologists at a single experienced center, and were used in clinical management. Secondly, the cohort included only patients with brain metastasis resection, corresponding to tumors typically ≥2 cm and frequently with solitary/oligometastatic disease, potentially limiting the results’ generalizability, though it is unclear whether HER2 expression heterogeneity is affected by metastasis size or multiplicity. Thirdly, we were unable to report precise IHC scores for all cases given some samples were no longer available for re-review, however, we mitigated this by excluding equivocal cases for each of the reported cutoffs. Finally, CNS response rates to T-DXd as a function of brain metastasis-specific HER2 expression levels has yet to be established.

HER2 expression varies substantially among patient-matched primary breast cancer and brain metastasis specimens, with the highest rate of discordance among patients with initially HER2-null disease. These findings identify a patient population that may benefit from T-DXd in the absence of previously-diagnosed HER2 expression. Correlates of HER2 dynamism and the potential role of metastasis sampling require further study in larger cohorts.

## Methods

### Data collection and analysis

A single-institution retrospective review was performed under the waiver of informed consent (given the retrospective nature of the data) granted by the Memorial Sloan Kettering Cancer Center IRB for patients with brain metastasis resection and sampling of ≥1 extracranial disease site (primary tumor, lymph node or extracranial metastasis). Clinical history and pathologic features were collected, including HER2 IHC score [PATHWAY anti-HER-2/neu (4B5; Ventana, Tucson, AZ); HercepTest (Dako, Carpinteria, CA)], fluorescence ISH (FISH; HER2/CEP17 ratio, HER2 CN) [HER2 IQFISH pharmDx (Dako); PathVysion HER-2 DNA Probe Kit (Vysis, Downers Grove, IL)], and *ERBB2* amplification status by next generation sequencing when available^15^. All IHC and ISH results were originally reported in accordance with contemporaneous ASCO/CAP breast cancer guideline recommendations. HER2 status was classified in this study as null/ undetectable (IHC 0), low (IHC 1 +, 1+ to 2 + /ISH-, or 2 + /ISH-) or positive (IHC 2 + /ISH +, or IHC 3 +). All cases without a granular IHC score were re-reviewed by a breast pathologist blinded to the patient’s other samples. FISH results were reclassified into 5 categories as defined by the 2018 ASCO/CAP guideline^16^. The presence of heterogeneity, defined as a second discrete population of amplified/non-amplified tumor cells, was noted if originally documented in IHC or FISH reports. Reports for IHC/ISH initially read at outside institutions were re-reviewed by a blinded breast pathologist. HER2 concordance rates were determined within patients and assessed using Cohen’s Kappa separately by the metastatic site (brain, lymph node, and extracranial). Among patients with a change from HER2-null primary breast cancer to HER2-expressing brain metastasis or HER2-expressing primary breast cancer to HER2-null brain metastasis, equality of proportions of gained versus lost expression were tested using the Rao-Scott Chi-squared test. A change in HER2 status from a primary breast cancer to a metastasis (either HER2-null primary breast cancer to a HER2-expressing metastasis or HER2-expressing primary breast cancer to a HER2-null metastasis) was modeled using logistic regression accounting for intrapatient variability. A post hoc sensitivity analysis was performed excluding patients with multiple and HER2 discordant primary breast tumors. A post hoc analysis on a random subset of 10 HER2 concordant (primary breast and brain metastasis pairs) and a random subset of 10 HER2 discordant (primary breast and brain metastasis pairs) was performed to understand treatment history distributions in relation to HER2 concordance/discordance. All tests were two-sided with a statistical level of significance set at <0.05. Analyses were performed in SAS v9.4 (SAS Institute, Cary, NC).

### Reporting summary

Further information on research design is available in the Nature Research Reporting Summary linked to this article.

### Supplementary information

Reporting Summary

## Data Availability

The datasets used and/or analyzed during the current study will be made available from the corresponding author on reasonable request.
